# Mechanistic Insights into the Inhibition of *Yersinia enterocolitica* Biofilm Formation by Lipoic Acid

**DOI:** 10.3390/microorganisms14030558

**Published:** 2026-02-28

**Authors:** Sichen Liao, Siqi Yang, Guoli Gong, Zhenbin Liu, Jiayi Zhang, Hongbo Li, Qing Sun, Haizhen Mo, Liangbin Hu, Lu Tian

**Affiliations:** 1 School of Food Science and Engineering, Shaanxi University of Science and Technology, Xi’an 710021, China; 200411031@sust.edu.cn (S.L.); 230412119@sust.edu.cn (S.Y.); guoligong@sust.edu.cn (G.G.); zhenbinliu@sust.edu.cn (Z.L.); lihongbo@sust.edu.cn (H.L.); mohz@sust.edu.cn (H.M.); hulb@sust.edu.cn (L.H.); 2 School of Food and Biological Engineering, Jiangsu University, Zhenjiang 212013, China; qing.sun@ujs.edu.cn

**Keywords:** lipoic acid, *Yersinia enterocolitica*, antibiofilm, transcriptomic

## Abstract

*Yersinia enterocolitica* is a foodborne pathogen that forms biofilms on surfaces, enhancing its survivability and increasing bacterial resistance, which poses a significant challenge to public health. Therefore, developing effective strategies to inhibit biofilm formation is crucial. Lipoic acid (LA) is a compound with antibiofilm properties. This study investigates the effects of LA on biofilm formation by *Y. enterocolitica* BNCC 108930 (a standard strain from the BeNa Culture Collection). Biofilm formation, maturation, removal, and cell viability were evaluated by crystal violet staining, extracellular polysaccharide assay, Methylthiazolyldiphenyl-tetrazolium bromide assays, motility, and quorum sensing (QS) assays. The results indicate that LA interferes with the early stages of biofilm formation by compromising cell membrane integrity and reducing cellular adhesion. Furthermore, 2.5 mg/mL of LA reduced biofilm biomass (with a 48 h treatment inhibition rate of 51.46 ± 1.29%) and extracellular polysaccharide production (with a relative inhibition rate of 30.09 ± 1.8%), while significantly reducing the metabolic activity of bacteria within the biofilm (inhibition rate over 85%) compared to the untreated group. Confocal laser scanning microscopy and field emission gun scanning electron microscopy confirm that LA induces a sparse biofilm structure, reduced aggregation, and decreased biofilm thickness to 21.33 ± 2.27 μm. Motility and QS assays demonstrate that LA affects flagellar motility and the secretion of N-acyl homoserine lactones. Transcriptome analysis revealed downregulation of genes involved in the QS system and biofilm formation (e.g., *lsrA*, *lsrC*, *lsrD*, *lsrR*, and *oppA*), as well as upregulation of genes related to bacterial chemotaxis and flagellar assembly (e.g., RS19655, RS15590, *fliE*, *fliJ*, *fliP*, *fliA*, and *fliK*). These alterations suggest that LA inhibits *Y. enterocolitica* biofilm formation by affecting intercellular communication and flagellar motility. This study highlights the antibiofilm properties of LA, providing a theoretical basis for potential applications in microbial and biofilm control.

## 1. Introduction

*Yersinia enterocolitica* is a facultative anaerobic Gram-negative bacterium with psychrophilic properties, capable of growth at refrigeration temperatures (4 °C). Consequently, it poses a significant risk during food storage and transportation under such conditions. As a zoonotic foodborne pathogen, *Y. enterocolitica* is prevalent in various food products, leading to severe health impacts, including intestinal infections such as gastroenteritis and enterocolitis [1,2]. Human infections commonly arise from consuming raw or undercooked pork, with less frequent occurrences linked to contaminated raw milk and untreated water [3]. Transmission primarily occurs through contaminated food or water, fecal-oral routes, and potentially person-to-person contact [4]. Additionally, foodborne pathogens can be transmitted among individuals [3]. *Y. enterocolitica* has emerged as a significant zoonotic pathogen in developed countries, being the third most commonly reported zoonotic pathogen in the European Union [5].

Given that microbial contamination is a major cause of foodborne disease outbreaks, *Y. enterocolitica* utilizes peritrichous flagellar motility to adhere to food contact surfaces (glass, stainless steel, plastic) and form biofilms, which is a major food safety concern [6]. Biofilms are microbial communities embedded in self-secreted extracellular polymeric substances (EPS) [7]. Their formation can be divided into four key stages: initial attachment, irreversible attachment, biofilm maturation, and biofilm dispersion. During the initial attachment stage, planktonic cells approach the material surface via reversible adhesion. Subsequently, bacteria secrete quorum-sensing signal molecules and an adhesive matrix, transitioning to irreversible attachment. In the maturation stage, cells proliferate extensively and secrete EPS, forming a stable three-dimensional architecture that enhances resistance to environmental stresses. Finally, portions of the population detach from the mature biofilm and re-enter the planktonic state, establishing a dynamic planktonic–biofilm cycle [8]. Studies have shown that biofilm formation by *Y. enterocolitica* is mediated by N-acyl homoserine lactones (AHLs) quorum-sensing (QS) signals and the second messenger cyclic di-guanosine monophosphate (c-di-GMP) [9]. The AHL synthase and receptor genes *yenI* and *yenR* facilitate QS signal transduction [10]. Cellular c-di-GMP levels are dynamically regulated by diguanylate cyclases (e.g., *ydaM*, *ardA*) and phosphodiesterases (e.g., *csrD*, *pdeH*); elevated c-di-GMP promotes synthesis of EPS, driving the transition from a planktonic to an adhesive state. In addition, the flagellar master regulator *flhDC* promotes initial surface colonization and adhesion of the biofilm by modulating flagellar motility and adhesive capacity [9]. Biofilm formation enhances bacterial protection and increases resistance to antibiotics and host immune responses, thereby complicating efforts to control the bacteria [11,12]. The food industry faces challenges as biofilm formation elevates bacterial resistance to various physical and chemical agents, potentially leading to cross-contamination during food processing, transportation, and storage. Thus, biofilm formation is a critical food safety concern [13].

Various strategies, including chemical preservatives, antibiotics, and advanced technologies such as pulsed electric fields and cold plasma treatments, have been employed to mitigate microbial biofilm contamination in food [14,15]. Among these approaches, natural antibiofilm compounds have garnered increased research interest. Lipoic acid (LA) is an organic acid with vitamin-like properties that can eliminate free radicals associated with accelerated aging and disease. It is primarily derived from sources such as spinach and broccoli and can be effectively absorbed through dietary intake [16]. Initially isolated from pig liver in 1951, LA is a natural product possessing both water-soluble and fat-soluble characteristics [17]. LA exhibits various beneficial effects as a coenzyme, including antibacterial, antioxidant, liver function enhancement, fatigue alleviation, dementia improvement, and cosmetic properties [18]. Its antioxidant capacity is reported to be 400 times greater than that of vitamins C and E, significantly surpassing that of currently popular compounds such as grape seed extract and green tea [19]. Furthermore, LA demonstrates potential for pharmaceutical and other therapeutic applications, and it has already been utilized in detoxification agents and diabetes treatment [20]. Studies have shown that LA exerts its antibacterial effect by causing excessive cell membrane permeability through membrane depolarization and reduced ATP synthesis [16,21]. However, research specifically examining the inhibitory effects of LA on *Y. enterocolitica* biofilm formation remains limited.

In this study, we investigated the inhibitory effects of LA on *Y. enterocolitica* biofilms at the cellular level. The impact of LA on biofilm formation and bacterial viability within biofilms was assessed using crystal violet staining (CVS), Methylthiazolyldiphenyl-tetrazolium bromide (MTT) assays, secretion of EPS, QS, confocal laser scanning microscopy (CLSM), and field emission gun scanning electron microscopy (FEG-SEM). To explore the specific mechanisms of LA, we further analyzed the expression changes in genes related to biofilm formation through transcriptomics, thereby providing insights into the molecular mechanism by which LA inhibits *Y. enterocolitica* biofilm formation. This study aims to provide a theoretical basis for the potential application of LA in microbial and biofilm control.

## 2. Materials and Methods

### 2.1. Reagents

**Figure 1:**
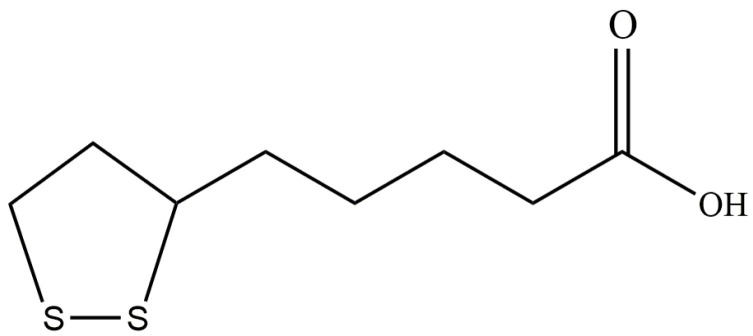
The chemical structure of Lipoic acid.

Lipoic acid (C_8_H_14_O_2_S_2_, HPLC ≥ 98%, CAS: 62-46-4) was purchased from Shanghai yuanye Bio-Technology Co., Ltd. (Shanghai, China), and its chemical structure is shown in Figure 1. It was dissolved in tryptic soy broth (TSB) and phosphate buffer (PBS) containing 3% ethanol, respectively. The 0 mg/mL of LA condition was defined as the vehicle control (TSB/PBS supplemented with 3% ethanol containing bacteria). Under the test conditions, the pH range of LA was 5.4–7.0 (Figure S1).

### 2.2. Strains and Cultures

*Y.enterocolitica* BNCC 108930 was purchased from the BeNa Culture Collection (BNCC, Beijing, China). *Chromobacterium violaceum* CV026 was purchased from Baosai Biotechnology (Baosai Biotech Inc. Co., Ltd., Shanghai, China). Single colonies were inoculated into TSB medium for activation, cultured in an incubator at 30 °C for 8 h (OD_600 nm_ = 0.5), and stored for later use.

### 2.3. Effect of LA on the Biofilm Formation of Y. enterocolitica BNCC 108930

We previously measured the minimum inhibitory concentration (MIC) of LA on *Y. enterocolitica* as 2.5 mg/mL [21], and to further investigate the minimum biofilm inhibitory concentration (MBIC) of LA on the biofilm formation of this species [22,23]. In total, 20 μL of *Y. enterocolitica* and 180 μL of LA solution prepared in TSB (0 mg/mL, 0.3125 mg/mL, 0.625 mg/mL, 1.25 mg/mL, and 2.5 mg/mL) were added into 96-well plates (Nunc, Roskilde, Denmark). TSB supplemented with 3% ethanol to inoculated bacteria was used as a negative control. Plates were incubated for biofilm formation for 12, 24, and 48 h. Biofilm biomass was quantified by the CVS method. After removal of the supernatant, 200 μL of PBS was added to each well and washed twice to remove non-adherent cells, followed by drying in an oven at 60 °C for 30 min. Then, 200 μL of 0.1% (*w*/*v*) crystal violet solution (Macklin Biochemical Co., Ltd., Shanghai, China) was added to each well and incubated for 30 min. Wells were subsequently washed twice with 200 μL of deionized water, and 200 μL of 33% (*v*/*v*) ice-cold acetic acid was added to each well to fully solubilize the stained biofilm. Absorbance was measured at 590 nm using a microplate reader (Varioskan Flash, Thermo Fisher, Waltham, MA, USA).

### 2.4. Removal of Mature Biofilms of Y. enterocolitica BNCC 108930 by LA

In total, 20 μL of *Y. enterocolitica* and 180 μL of TSB were added to a 96-well plate (Nunc, Roskilde, Denmark) and incubated at 30 °C for 48 h to form mature biofilms. Following incubation, the mature biofilm was washed 2–3 times with 200 μL of sterile PBS, and varying concentrations of LA prepared in PBS (0 mg/mL, 0.625 mg/mL, 1.25 mg/mL, and 2.5 mg/mL) were introduced. PBS supplemented with 3% ethanol to inoculated bacteria was used as a negative control. The plates were then incubated at 30 °C for an additional 24 h. The efficacy of LA in eradicating the mature biofilm was assessed using the CVS method.

### 2.5. Determination of Extracellular Polysaccharides of Y. enterocolitica BNCC 108930 Biofilms

The effect of LA on the extracellular polysaccharide content of *Y. enterocolitica* during biofilm formation was assessed using ruthenium red staining [24]. Following the cultivation method described in Section 2.3, *Y. enterocolitica* biofilms were treated with varying concentrations of LA prepared in TSB (0 mg/mL, 0.3125 mg/mL, 0.625 mg/mL, 1.25 mg/mL, and 2.5 mg/mL) and incubated at 30 °C for 48 h. TSB supplemented with 3% ethanol to inoculated bacteria was used as a negative control. After discarding the supernatant, 200 μL of a 0.01% (m/v) ruthenium red (CAS: 11103-72-3, Bioengineering Co., Ltd., Shanghai, China) staining solution was added, and the samples were incubated at 30 °C for 60 min. Subsequently, the absorbance of each well at OD_450nm_ was measured using a microplate reader (Varioskan Flash, Thermo Fisher, Waltham, MA, USA).

### 2.6. Determination of Proteins in Y. enterocolitica BNCC 108930 EPS

The Bicinchoninic acid (BCA) protein assay operates on the principle that proteins can reduce Cu^2+^ to Cu^+^, resulting in the formation of a purple complex with BCA, which facilitates the quantitative detection of protein content. The biofilm of *Y. enterocolitica* was treated with varying concentrations of LA prepared in TSB (0 mg/mL, 0.3125 mg/mL, 0.625 mg/mL, 1.25 mg/mL, and 2.5 mg/mL), following the cultivation method described in Section 2.3. TSB supplemented with 3% ethanol to inoculated bacteria was used as a negative control. After incubation at 30 °C for 48 h, excess medium and planktonic cells were removed, and the biofilms were rinsed twice with 200 μL of sterile PBS. Subsequently, 200 μL of PBS was added to each well, and the attached biofilm cells were collected by pipetting repeatedly, followed by centrifugation at 5000× *g* for 10–15 min at 4 °C to obtain the supernatant. Finally, the supernatant was used to quantify the protein content within the biofilm EPS by the BCA protein assay kit (Sangon, Shanghai, China) [25].

### 2.7. Determination of Metabolic Activity in the Biofilms of Y. enterocolitica BNCC 108930

The MTT assay relies on the conversion of living cells into formazan crystals to evaluate cell viability [26]. The effect of LA on metabolic activity in mature *Y. enterocolitica* biofilm was determined by MTT assay. Mature *Y. enterocolitica* biofilms were cultured as described in Section 2.4. Subsequently, LA solutions at varying concentrations prepared in PBS (0 mg/mL, 0.625 mg/mL, 1.25 mg/mL, and 2.5 mg/mL) were used to treat the biofilms at 30 °C for 1 and 2 h, respectively. PBS supplemented with 3% ethanol to inoculated bacteria was used as a negative control. Then, 200 μL of MTT (0.5 mg/mL) was added to each well and incubated at 30 °C for 4 h. After removing MTT and excess medium, the formazan crystals were dissolved in 100 μL of DMSO. The optical density (OD) at 570 nm was measured using a microplate reader (Varioskan Flash, Thermo Fisher, Waltham, MA, USA).

### 2.8. CLSM Observations

CLSM was employed to assess the inactivation of *Y. enterocolitica* cells in biofilms by LA. Biofilms were cultured in a 24-well plate (Nunc, Copenhagen, Denmark) with sterile glass slides. Utilizing the method described in Section 2.3, 1800 μL of different concentrations of LA prepared in TSB (0 mg/mL, 0.625 mg/mL, 1.25 mg/mL, and 2.5 mg/mL) were added along with 200 μL of *Y. enterocolitica*, and the mixture was incubated at 30 °C for 48 h. TSB supplemented with 3% ethanol to inoculated bacteria was used as a negative control. Subsequently, 3 μL of a PI/SYTO 9 (1:1, final concentration of 1 μM each) staining mixture (LIVE/DEAD BacLight™ Bacterial Viability Kit, Molecular Probes, Thermo Fisher, Waltham, MA, USA) was added per mL and incubated in the dark for 15 min. Finally, the stained biofilms were observed using CLSM (LSM800, Carl Zeiss, Yarra, Germany).

To evaluate the cultivability of *Y. enterocolitica* biofilms after LA treatment, the method described in Section 2.6 was used to collect the biofilms into centrifuge tubes, followed by serial dilution with PBS and plating on TSB agar plates. These plates were then incubated at 30 °C for 12 h for colony counting.

### 2.9. FEG-SEM Observations

Following the cultivation method outlined in Section 2.8, *Y. enterocolitica* biofilms were treated with varying concentrations of LA prepared in TSB (0 mg/mL, 0.625 mg/mL, 1.25 mg/mL, and 2.5 mg/mL). TSB supplemented with 3% ethanol to inoculated bacteria was used as a negative control. After incubating at 30 °C for 48 h, the influence of LA on *Y. enterocolitica* biofilm formation was examined using FEG-SEM (MLA 650, FEI, Hillsboro, OR, USA), as described by Fan et al. [22].

### 2.10. Effect of LA on the Motility of Y. enterocolitica BNCC 108930

Motility was determined according to published methods [26,27]. The *Y. enterocolitica* cell pellets treated with LA prepared in PBS (0 mg/mL, 0.625 mg/mL, 1.25 mg/mL, and 2.5 mg/mL) for 2 h were collected, washed 2–3 times with PBS, and resuspended in 1 mL of PBS. PBS supplemented with 3% ethanol to inoculated bacteria was used as a negative control. The OD value at 600 nm was measured using a microplate reader (Varioskan Flash, Thermo Fisher, Waltham, MA, USA). Subsequently, A 1 μL culture was inoculated at the center of TSB semi-solid agar plates containing 0.3% agar, and the plates were incubated at 30 °C for 24 h. Motility was then observed and photographed.

### 2.11. Effect of LA on QS of Y. enterocolitica BNCC 108930

A bacterial suspension of *C. violaceum* CV026 was prepared according to the protocol outlined in Section 2.2. The semi-solid TSB medium containing 1% agar was mixed with the overnight cultured suspension of *C. violaceum* CV026 (OD_600nm_ = 1.0) in a 1:1 ratio and immediately poured onto Petri plates. *Y. enterocolitica* was treated with LA prepared in PBS (0 mg/mL, 0.625 mg/mL, 1.25 mg/mL, and 2.5 mg/mL) for 2 h. PBS supplemented with 3% ethanol to inoculated bacteria was used as a negative control. Subsequently, 1 μL of the culture was inoculated onto semi-solid agar plates containing TSB medium mixed with *C. violaceum* CV026 suspension and incubated at 28 °C for 24 h. The QS inhibitory effect of LA on *Y. enterocolitica* is defined by the absence or reduction in the violacein halo (diameter in millimeters) [27,28]. In addition, the OD value at 600 nm of *C. violaceum* CV026 treated with LA (0 mg/mL, 0.625 mg/mL, 1.25 mg/mL, and 2.5 mg/mL) for 2 h was measured using a microplate reader (Varioskan Flash, Thermo Fisher, Waltham, MA, USA).

### 2.12. Transcriptomics

According to the cultivation method described in Section 2.3, *Y. enterocolitica* biofilms were treated with LA prepared in TSB (0 mg/mL, 2.5 mg/mL) and incubated at 30 °C for 48 h. Five biological replicates were set up for both the control and treatment groups. After the incubation period, the bacterial pellets were collected by centrifugation. Total RNA was extracted using the RNAprep Pure Total RNA Extraction Kit (TransGen Biotech. Co., Ltd., Beijing, China). RNA purity was assessed using a NanoDrop 2000c spectrophotometer (Thermo Fisher Scientific, Waltham, MA, USA), with an A_260_/A_280_ ratio of 1.8–2.1 indicating high RNA purity, and RNA integrity was measured using a Bioanalyzer 2100 (Agilent Technologies, CA, USA) with RIN > 7 indicating stable RNA expression. After confirming RNA quality, ribosomal RNA was removed from the samples using the Ribo-Zero rRNA Removal Kit (Epicentre Biotechnologies, WI, USA), and purified RNA was used to construct cDNA libraries following the NEBNext^®^ Ultra^TM^ II Directional RNA Library Prep Kit (Illumina, CA, USA) instructions. Subsequently, high-throughput sequencing was conducted on the Illumina NovaSeq platform. To ensure the quality of data analysis, raw data were quality-controlled using Fastp v0.23.4 software to remove low-quality reads, followed by sequence alignment using Bowtie2 v2.5.3 software to obtain clean reads (using *Yersinia enterocolitica* subsp. palearctica Y11 as the reference genome, https://www.ncbi.nlm.nih.gov/datasets/genome/GCF_000253175.1/, accessed on 1 April 2022). Based on gene length and read counts, fragments per kilobases per million reads (FPKM) were calculated using RSEM v1.3.3 software to estimate the expression level of each gene. Differentially expressed genes (DEGs) between treated and control samples were then statistically analyzed using the DESeq R package (v1.18.0). DEGs were selected based on the criteria |log_2_ (fold change)| ≥ 1 and false discovery rate (FDR) ≤ 0.05. Principal component analysis (PCA), volcano plots, and heatmaps were generated using R software (v4.2.2) to evaluate grouping trends among different samples. Finally, functional analysis of these DEGs was conducted using the GO (http://geneontology.org/, accessed on 24 April 2022) and KEGG (http://www.genome.jp/kegg/, accessed on 24 April 2022) databases.

The raw sequence data of the transcriptome have been uploaded to the National Center for Biotechnology Information (NCBI) GenBank, with the BioProject ID PRJNA1369180.

### 2.13. Real-Time Quantitative Polymerase Chain Reaction (RT-qPCR)

To validate the transcriptome data, we randomly selected 10 DEGs for RT-qPCR analysis. The experiment followed the protocol of Tian et al. [29], using RealUniversal Color Fluorescent Quantitative Premix (SYBR Green) (Tiangen Biotech Co., Beijing, China) and the 2^−ΔΔCt^ method to calculate gene expression levels. Primer sequences are provided in Table S1.

### 2.14. Statistical Analysis

All data were expressed as mean ± SD and were analyzed using SPSS 19.0 software based on biological replicates. For data with a normal distribution, one-way analysis of variance (ANOVA) and Tukey’s test were used for intergroup comparisons, and further Student’s t-test was used to verify differences between groups. A *p*-value of less than 0.05 indicated a statistically significant difference.

## 3. Results

### 3.1. Effect of LA on the Biofilm Formation of Y. enterocolitica BNCC 108930

**Figure 2:**
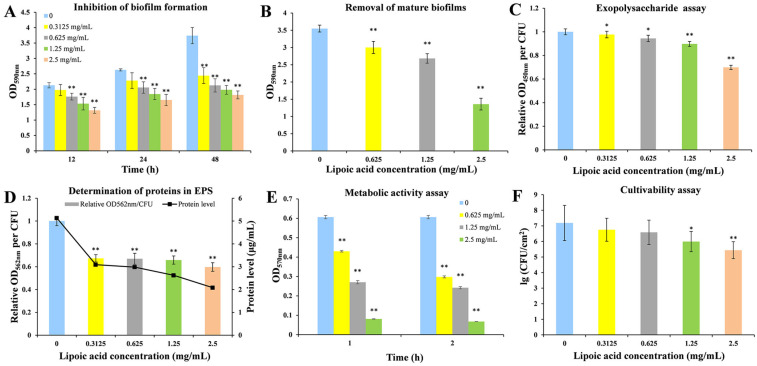
(**A**) The CVS method was used to determine the inhibition of LA on biofilm formation of *Y.enterocolitica*. (**B**) Removal effect of LA on mature biofilms of *Y.enterocolitica* (formed for 48 h). (**C**) Effects of LA on exopolysaccharide secretion in *Y.enterocolitica* EPS. (**D**) Effects of LA on protein secretion in *Y.enterocolitica* EPS. (**E**) Effect of LA on metabolic activity in *Y.enterocolitica* biofilm. (**F**) Effect of LA on the cultivability of *Y. enterocolitica* biofilms. Values are presented as the means of independent triplicate measurements (*n* = 3, representing biological replicates). * *p* < 0.05, ** *p* < 0.01.

We investigated the effects of LA on biofilm formation of *Y. enterocolitica* at different treatment durations using the CVS method. As shown in Figure 2A, compared to the untreated control group, the inhibition rates of biofilm formation for *Y. enterocolitica* after treatment with 0.625 mg/mL, 1.25 mg/mL, and 2.5 mg/mL of LA for 12 h were 17.27 ± 1.10% (*p* < 0.01), 28.03 ± 2.06% (*p* < 0.01), and 38.24 ± 0.95% (*p* < 0.01), respectively. Notably, at a concentration of 2.5 mg/mL, the inhibitory effect of LA on *Y. enterocolitica* biofilm formation remained effective over an extended formation period. When the treatment duration was prolonged to 48 h, the inhibitory effect was most pronounced, achieving an inhibition rate of 51.46 ± 1.29% (*p* < 0.01). Therefore, LA exhibits a significant inhibitory effect on the biofilm formation of *Y. enterocolitica*, which is dose-dependent. On this basis, the MBIC_50_ of LA against *Y. enterocolitica* was determined to be 2.5 mg/mL, and this concentration with a 48 h exposure was selected for subsequent experiments.

### 3.2. The Removal Effect of LA on Mature Biofilms of Y. enterocolitica BNCC 108930

Biofilms provide physical protection for bacteria and confer significant resilience to environmental stressors, making the effective eradication of these biofilms a considerable challenge. Therefore, we evaluated the efficacy of LA in removing mature biofilms formed by *Y. enterocolitica*. As shown in Figure 2B, the biofilm biomass in the untreated group had an OD_590nm_ of 3.55. Treatment with LA at concentrations of 0.625 mg/mL, 1.25 mg/mL, and 2.5 mg/mL resulted in biofilm removal rates of 15.41 ± 1.81% (*p* < 0.01), 24.37 ± 2.6% (*p* < 0.01), and 61.72 ± 1.64% (*p* < 0.01), respectively. The observed differences at varying concentrations indicate that increasing LA concentrations correlate with a reduction in residual biofilm biomass. This indicates that LA not only inhibits the early formation of biofilms by *Y. enterocolitica* but also effectively removes mature biofilms.

### 3.3. Effect of LA on Exopolysaccharides in the Biofilms of Y. enterocolitica BNCC 108930

Exopolysaccharide production was measured to reflect extracellular polymer secretion. Ruthenium red is used as a cationic dye to determine the content of exopolysaccharides. Figure 2C shows the effect of LA on exopolysaccharide production per bacterial cell in *Y. enterocolitica* biofilm formed for 48 h. Treatment with 0.625 mg/mL of LA resulted in a 5.6 ± 2.7% reduction in relative exopolysaccharide production (*p* < 0.05). The relative production of exopolysaccharide decreased in a concentration-dependent manner, reaching a 30.09 ± 1.8% reduction at 2.5 mg/mL of LA (*p* < 0.01). These results indicate that LA exerts a significant inhibitory effect on exopolysaccharide production within *Y. enterocolitica* biofilms.

### 3.4. Effect of LA on Extracellular Protein in Y. enterocolitica BNCC 108930 EPS

As illustrated in Figure 2D, after 48 h of biofilm formation by the control group of *Y. enterocolitica*, the protein content in the EPS was measured to be 5 μg/mL. Following treatment with LA, the protein content in the EPS of *Y. enterocolitica* decreased, and with increasing concentrations of LA, the relative protein levels in the EPS gradually diminished. Under treatment with 2.5 mg/mL of LA, the protein content in the EPS of *Y. enterocolitica* was 2 μg/mL, representing a 59.42 ± 3.74% decrease compared with the control group (*p* < 0.01). This indicates that LA significantly affects the secretion of proteins in the EPS of *Y. enterocolitica*.

### 3.5. Effect of LA on Metabolic Activity in the Biofilms of Y. enterocolitica BNCC 108930

For most cell populations, the amount of formazan produced correlates with the number of viable cells, making MTT assays widely utilized to measure the effects of drugs on cellular activity. As illustrated in Figure 2E, treatment durations of 1 h and 2 h with LA significantly decreased the cell viability of *Y. enterocolitica* in biofilms. After 1 h of treatment with LA (0.625 mg/mL, 1.25 mg/mL, and 2.5 mg/mL), the survival rates of *Y. enterocolitica* in the biofilms were reduced by 29.11 ± 0.41% (*p* < 0.01), 55.4 ± 0.85% (*p* < 0.01), and 86.65 ± 0.1% (*p* < 0.01), respectively, compared to the control group. When the exposure time was extended to 2 h, the metabolic activity of *Y. enterocolitica* in biofilms treated with LA decreased significantly by 50.87 ± 0.52% (*p* < 0.01), 60.09 ± 0.58% (*p* < 0.01), and 88.84 ± 0.03% (*p* < 0.01), respectively. These experimental results indicate that LA exerts a notable inhibitory effect on metabolic activity in biofilms.

### 3.6. CLSM Observation of the Effect of LA on Bacterial Activity in the Biofilms of Y. enterocolitica BNCC 108930

**Figure 3:**
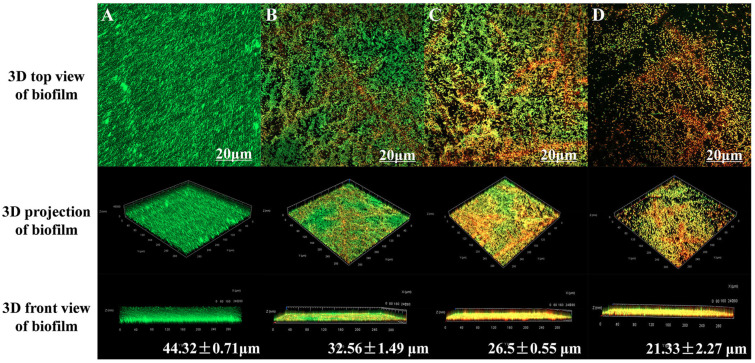
CLSM observation of the effect of LA on *Y. enterocolitica* biofilms after 48 h of treatment. (**A**) untreated, (**B**) 0.625 mg/mL LA, (**C**) 1.25 mg/mL LA, and (**D**) 2.5 mg/mL LA. Values are presented as the means of independent triplicate measurements (*n* = 3, representing biological replicates).

The membrane-permeability double-staining method is commonly used to assess bacterial viability. SYTO 9, a membrane-permeant nucleic acid dye, can traverse intact cell membranes and label all bacteria with green fluorescence, whereas PI, owing to its larger molecular size and positive charge, can only enter cells with compromised membrane integrity and, by virtue of its higher nucleic acid-binding affinity, competitively displaces SYTO 9 to produce a red fluorescence signal. By examining the three-dimensional architecture of the biofilms, we more clearly delineated the effects of LA on the viability of bacteria within *Y. enterocolitica* biofilms and on biofilm thickness. As shown in Figure 3 and Figure S2, the control exhibited intense, dense green fluorescence and a biofilm thickness of 44.32 ± 0.71 μm, indicating an intact biofilm structure and preserved intrabiofilm bacterial viability. With increasing concentrations of LA, the *Y. enterocolitica* biofilms became sparser and biofilm thickness decreased markedly. Relative to the control, LA at 0.625 and 1.25 mg/mL increased the proportion of PI fluorescence to 30.72 ± 1.88% and 40.53 ± 1.32%, respectively (*p* < 0.01), and reduced biofilm thickness to 26.53 ± 3.55% and 40.19 ± 2.16%, respectively (*p* < 0.01). Notably, treatment with 2.5 mg/mL of LA produced the strongest red fluorescence across the field, with PI-derived fluorescence comprising 63.48 ± 1.21% of the signal, and reduced biofilm thickness to 21.33 ± 2.27 μm. At the same time, the growth of *Y. enterocolitica* was reduced by about 1.75 lg CFU/cm^2^ compared to the untreated group (*p* < 0.01) (Figure 2F). These findings indicate that LA strongly reduces biofilm thickness and CFU counts of *Y. enterocolitica*.

### 3.7. FEG-SEM Observation of the Effect of LA on Biofilm Formation of Y. enterocolitica BNCC 108930

**Figure 4:**
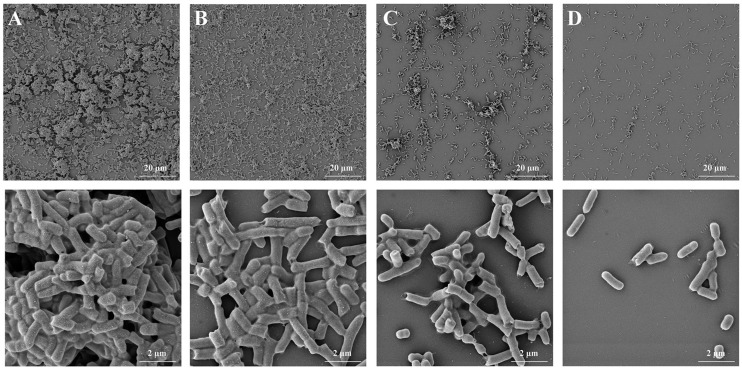
FEG-SEM observation of the effect of LA on *Y. enterocolitica* biofilms after 48 h of treatment. (**A**) untreated, (**B**) 0.625 mg/mL LA, (**C**) 1.25 mg/mL LA, and (**D**) 2.5 mg/mL LA.

To confirm the role of LA, we employed FEG-SEM to observe and evaluate the effects of LA on the biofilm formation of *Y. enterocolitica*. The results, presented in Figure 4, indicate that untreated *Y. enterocolitica* biofilms were compact, with cells forming clusters. After treatment with 0.625 mg/mL of LA, the biofilms became sparse, and the bacterial clusters diminished. Treatment with 1.25 mg/mL of LA, the number of *Y. enterocolitica* cells decreased significantly, making it difficult to observe bacterial aggregation. When the biofilm was exposed to 2.5 mg/mL of LA, it became even more sparse, and no bacterial clusters were observed. These findings indicate that LA has a significant inhibitory effect on the biofilm of *Y. enterocolitica*.

### 3.8. Effect of LA on the Motility of Y. enterocolitica BNCC 108930

**Figure 5:**
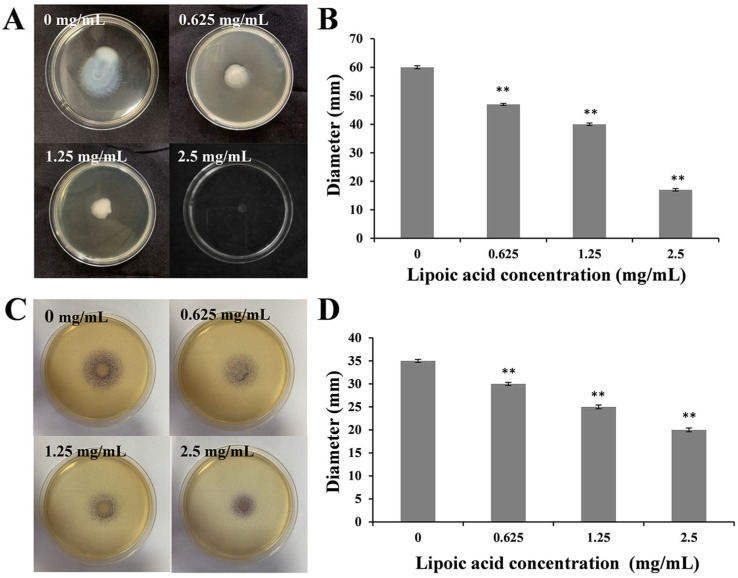
Effect of LA on the motility and QS of *Y.enterocolitica*: (**A**) Motility image and (**B**) Motility diameter; (**C**) Plate image of QS inhibition and (**D**) Violacein diameter. Values are presented as the means of independent triplicate measurements (*n* = 3, representing biological replicates). ** *p* < 0.01.

*Y. enterocolitica* possesses periflagellar flagella, which confer motility to the bacterium. Figure 5A,B show that the motility diameter of *Y. enterocolitica* in the control group was 60 ± 0.5 mm. After treatment with LA at concentrations of 0.625 mg/mL, 1.25 mg/mL, and 2.5 mg/mL, the motility diameter decreased by 21.67 ± 1.6%, 33.33 ± 1.42%, and 71.67 ± 1.76%, respectively (*p* < 0.01). To confirm that the inhibition of motility was not affected by a reduction in bacterial numbers, we standardized the initial inoculum at the time of inoculation to CFU (Figure S3). The results showed that with increasing LA concentrations, motility was significantly reduced even when CFU decreased, further supporting that LA can effectively inhibit *Y. enterocolitica* motility mediated by periflagellar flagella.

### 3.9. Effect of LA on QS of Y. enterocolitica BNCC 108930

The production of violacein by *C. violaceum* is regulated by the QS system, which synthesizes AHLs through the action of the autoinducer synthase *cviI*. We utilized the *C. violaceum* mutant strain *C. violaceum* CV026 to investigate the effect of LA on the synthesis of AHL autoinducers. As illustrated in Figure 5C,D and Figure S4, with increasing concentrations of LA, the diameter of the violacein halo decreased progressively, while not inhibiting the growth of *C. violaceum*. Treatment with 2.5 mg/mL of LA reduced the violacein halo diameter by 42.86 ± 0.87% (*p* < 0.01) compared to the control group. This reduction is consistent with inhibition of violacein production, and colony size was smaller at 2.5 mg/mL.

### 3.10. Transcriptomics

#### 3.10.1. Global Analysis of Transcriptomics

**Figure 6:**
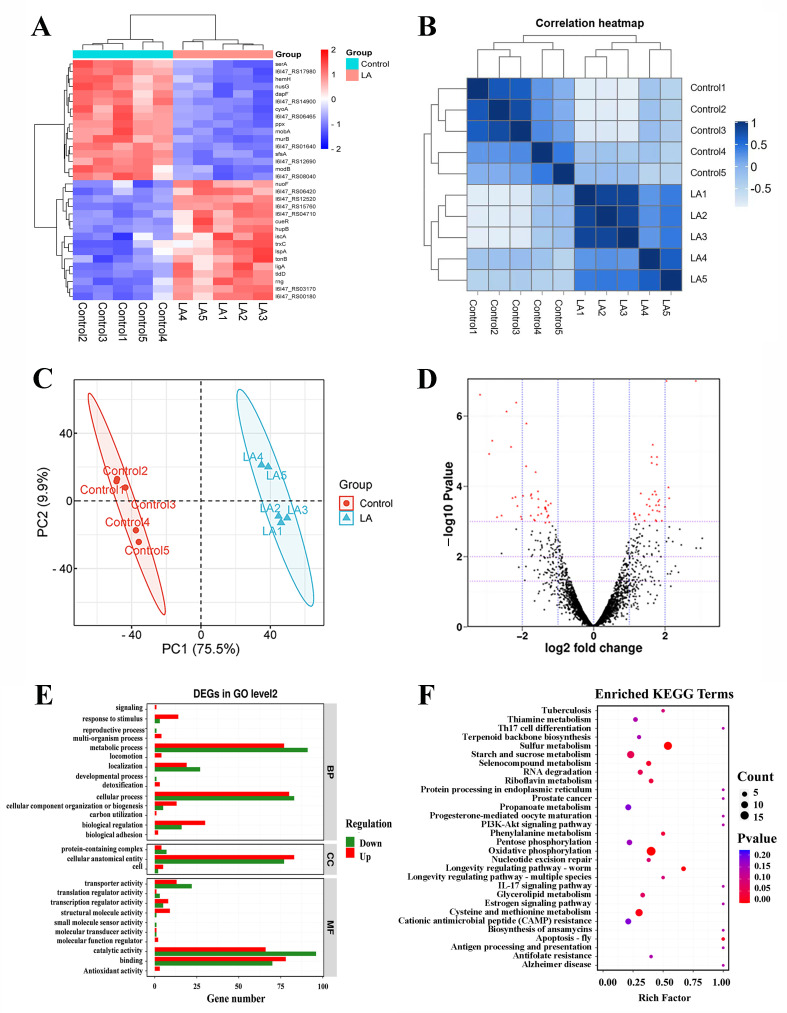
Transcriptomic analysis of *Y. enterocolitica* biofilms treated with 2.5 mg/mL of LA for 48 h. (**A**) Clustering heat map of DEGs (red represents upregulation, and blue represents downregulation). (**B**) Gene expression distance heat map. (**C**) PCA. (**D**) Volcano map of DEGs. (**E**) GO annotation map of DEGs. (**F**) KEGG enrichment map.

To thoroughly investigate the comprehensive effects of LA on *Y. enterocolitica*, we performed RNA sequencing of *Y. enterocolitica* treated with LA at MIC. Hierarchical clustering analysis of the relationships between samples and genes was conducted based on overall gene expression levels (Figure 6A), accompanied by a heatmap depicting hierarchical clustering according to gene expression levels (Figure 6B). The results indicated that the gene expression levels underwent significant changes following LA treatment. Furthermore, PCA converts complex information into a composite indicator that reflects the patterns of the original data. The tens of thousands of dimensions of information contained in a sample are downscaled into a composite indicator of several dimensions to facilitate comparisons between samples while ensuring that as much of the information contained in the original data is retained as possible. The PCA illustrates that the scatter distribution between the experimental and control groups is far apart, indicating significant differences (Figure 6C). Additionally, the volcano plot demonstrated that LA induced the upregulation of 259 DEGs and the downregulation of 220 DEGs in *Y. enterocolitica* (upregulated expression on the left and downregulated expression on the right) (Figure 6D).

GO database can be used to classify and annotate DEGs and pathways affected by gene-encoded proteins (Figure 6E). In biological processes, DEGs were classified into metabolic process, cellular process, and biological regulation. Among the other GO functions of the cellular components type, most are composed of cellular anatomical entities. In molecular function types, the role of DEGs was mainly focused on binding and catalytic activity. The KEGG database was used to further analyze the metabolic function of DEGs, and DEGs were located in specific biological pathways. KEGG pathway analysis showed that the DEGs in *Y. enterocolitica* cells after LA treatment were significantly involved in metabolic pathways such as oxidative phosphorylation, sulfur metabolism, starch and sucrose metabolism, cysteine and methionine metabolism (Figure 6F).

#### 3.10.2. Validation of DEGs by RT-qPCR

Ten genes (*nuoH*, *nuoI*, *cyoE*, *carA*, *RS19655*, *lsrA*, *lsrC*, *cysM*, *cysW*, and *metK*) were randomly selected for RT-qPCR validation. The trends of these 10 genes were consistent between the RT-qPCR and RNA-seq results (Figure S5), indicating that the detection results are reliable and accurate.

## 4. Discussion

*Y. enterocolitica* is a pathogen well-known for its role in foodborne diseases and holds significant public health implications. In recent years, biofilm formation has become a key factor in the persistence and virulence of bacterial pathogens [30]. This study highlights the impact of LA on biofilm formation by *Y. enterocolitica* and provides insights into its underlying mechanisms.

The results of this study indicate that LA can significantly inhibit biofilm formation by *Y. enterocolitica* (MBIC_50_ of 2.5 mg/mL), especially during the early stages of biofilm development. The mechanism behind this effect may involve LA disrupting the structural and functional integrity of the *Y. enterocolitica* biofilm, thereby affecting cellular adhesion and stability during biofilm formation [28]. Research indicates that the MBIC of oliverine, guatterine, liriodenine, oliveridine, and pachypodanthine against *Y. enterocolitica* were 25 μmol/L, 100 μmol/L, 25 μmol/L, 25 μmol/L, and 12.5 μmol/L, respectively [28]. Furthermore, 64 µg/mL of equol significantly reduced biofilm formation by 61.3% in *Y. enterocolitica* ATCC 9610 [31]. Camellia saponins exhibited significant inhibition of biofilms of *B. cereus* ATCC 10987 and ATCC 14579, with an MBIC of 64 mg/mL [32]. In comparison, LA’s ability to inhibit biofilm formation in *Y. enterocolitica* was found to be at a favorable level. It is noteworthy that although LA exhibits inhibitory activity against *Y. enterocolitica* at 2.5 mg/mL, a subset of cells can complete initial surface attachment before the full inhibitory effect is realized. Biofilm formation and bacterial adaptive responses may confer tolerance to LA among biofilm-embedded cells, resulting in measurable accumulation of biofilm biomass even at concentrations at or near the MIC. Mature biofilms provide bacteria with a protective barrier, enabling survival in adverse environments and enhancing antimicrobial resistance [33]. We found that LA effectively eradicates mature biofilms. As the LA concentration increases, the residual biofilm biomass of *Y. enterocolitica* decreases, which is consistent with the report of Liao et al. [34]. Furthermore, the EPS of biofilms is primarily composed of extracellular polysaccharides, proteins, and DNA, which are crucial for forming and maintaining biofilm structure [35]. After LA treatment, the polysaccharide content of the EPS in *Y. enterocolitica* biofilms significantly decreased, and this directly impacted the biofilm’s structural integrity and survival ability. Studies have shown that EPS is closely related to cell adhesion and stress resistance [36], suggesting that LA’s inhibitory effect on biofilm formation may be mediated through interference with the function and synthesis of EPS. Notably, LA can simultaneously affect the secretion of both polysaccharides and proteins, which are essential components of biofilm structure. It has been previously reported that carvacrol can effectively inhibit the secretion and production of extracellular polysaccharides and proteins of *Listeria monocytogenes* [37]. FEG-SEM observations further confirmed that LA-treated biofilms were sparse, with a noticeable reduction in bacterial aggregation, and the same results were observed in the study by Liu et al. [35]. CLSM observations provided additional insights, showing that LA strongly reduces biofilm thickness and CFU counts of *Y. enterocolitica*, which may be due to LA-induced damage to the *Y. enterocolitica* cell membrane, leading to decreased bacterial viability within the biofilm. The study also found that LA weakens the motility of *Y. enterocolitica*, which may inhibit bacterial migration and biofilm formation, thereby reducing the pathogen’s ability to invade. QS is a communication mechanism based on bacterial population density that relies on small diffusible signal molecules to transmit information [38]. Among these, AHLs are the core signaling molecules of QS, regulating the collective behavior of bacterial populations [39]. LA may affect the synthesis and degradation of signaling molecules by disrupting bacterial communication, thereby inhibiting biofilm formation. Similarly, consistent with the findings of Kamli et al., oregano extract also inhibited the production of violacein by *C. violaceum* [40].

**Figure 7:**
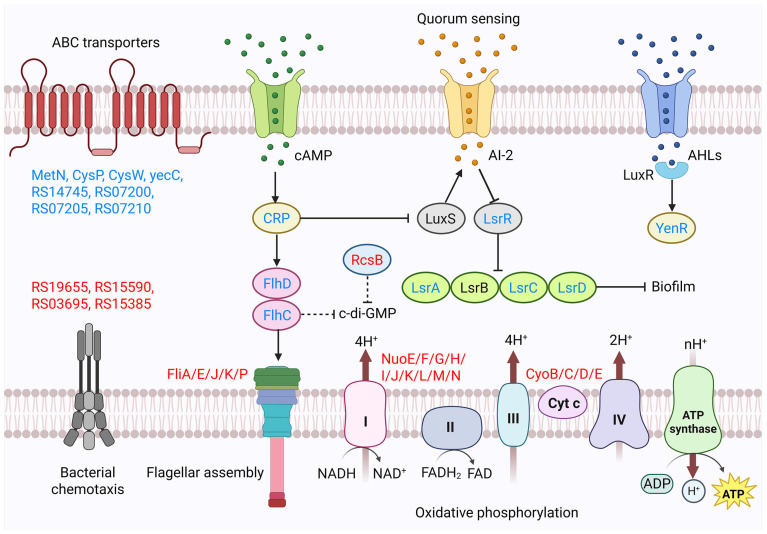
Proposed model: In *Y. enterocolitica* biofilms treated with LA, gene expression changes occur in pathways related to energy metabolism, QS systems, and flagellar motility (upregulated genes are highlighted in red, downregulated genes are highlighted in blue).

Molecular-level analysis further reveals that LA may inhibit the growth and biofilm formation of *Y. enterocolitica* by affecting pathways related to amino acid metabolism, energy metabolism, the QS system, and flagellar motility (Table 1 and Figure 7). Our data suggest that cysteine and methionine metabolism, and arginine biosynthesis exhibit varying degrees of changes (e.g., *cysK*, *metK*, *argB*, *argC*, and *argF*), indicating that LA may lead to intracellular osmotic homeostasis imbalances by disrupting the synthesis and metabolic pathways of amino acids [41]. Notably, multiple genes associated with oxidative phosphorylation are upregulated (e.g., *nuoF*, *nuoI*, *nuoJ*, *nuoH*, *nuoG*, *cyoE*, and *cyoB*). Wu et al. [42] demonstrated that the genes of the *Nuo* cluster encode NADH dehydrogenase. NADH dehydrogenase plays a pivotal role in cellular anabolism and the maintenance of intracellular redox balance. The up-regulation of NADH dehydrogenase genes may suggest that *Y. enterocolitica* requires increased energy production to maintain biochemical reactions and normal physiological activities within the cell, thereby enabling the organism to counteract stimuli induced by environmental changes [43]. Fatty acid metabolism is an important pathway for cellular energy production. In *Y. enterocolitica* cells treated with LA, the genes *fabD*, *fabF*, and *fabZ*, which are involved in fatty acid synthesis and metabolism, were down-regulated. Among them, *fabD* is a monomeric protein that accepts the malonyl moiety from malonyl-CoA to form a more stable malonyl-serine intermediate [44]. *fabF*, located in the fatty acid synthesis gene cluster, influences fatty acid synthesis [45]. The down-regulation of genes involved in fatty acid metabolism may indicate insufficient fatty acid breakdown and energy supply. In addition, ABC transporters are mainly involved in the uptake of trace elements and nutrients through medium and high-affinity pathways [46,47]. Notably, sulfur is a vital component of intracellular biotin, coenzyme A, and glutathione, and the presence of these substances is essential for maintaining cellular life activities. The down-regulation of *cysP* and *cysW* may impair the binding and penetration of these sulfur-containing coenzymes, potentially disrupting relevant physiological activities and weakening cellular functions [48]. The *luxI*/*luxR*-type autoinduction system mediated by AHLs functions as an intraspecific signaling system in Gram-negative bacteria. The transcript encoding the *luxR*-family transcriptional regulator *yenR* was downregulated, indicating that LA may inhibit the QS system by suppressing AHLs synthesis and thereby reducing biofilm formation by *Y. enterocolitica* [9]. This is consistent with the findings of Gao et al. [9]. In addition, autoinducer-2 (AI-2) is considered a universal interspecies signal. Our data showed that the expression of genes encoding the autoinducer-2 ABC transporter ATP-binding protein *lsrA*, the AI-2 transport system permease *lsrC*, the autoinducer-2 ABC transporter permease *lsrD*, and the transcriptional regulator *lsrR* (*lsrA*, *lsrC*, *lsrD*, and *lsrR*) was downregulated. *lsrC* is a transmembrane protein that forms part of the membrane channel; its downregulation may suggest decreased binding and permeability of AI-2 transporters, reduced synthesis of membrane channels, and impaired transmembrane transport [49]. Furthermore, expression of the oligopeptide transport substrate-binding protein *oppA*, which is associated with QS, was downregulated; this may directly suppress bacterial motility, adhesion, and biofilm formation [50]. Fang et al. [51] reported that *rcsB* acts as an inhibitor of biofilm formation, capable of preventing biofilm development and the accumulation of c-di-GMP. Moreover, the gene *CRP*, which encodes the cAMP receptor protein, influences flagellar synthesis and biofilm formation by modulating c-di-GMP levels [52]. *CRP* positively regulates transcription of *flhDC*, thereby mediating flagellum-related physiological processes. Previous reports have shown that protocatechuic acid affects *Y. enterocolitica* biofilm formation by reducing *flhDC* expression [9], which aligns with our results. As the principal organelles of bacterial motility, flagella play critical roles in locomotion, chemotaxis, biofilm formation, and environmental sensing. When bacteria perceive adverse external conditions, they employ flagellar-mediated motility to elicit directional responses to gradients of environmental factors, moving toward favorable stimuli and away from harmful ones [53]. In our study, genes involved in bacterial chemotaxis (RS19655, RS15590, RS03695, RS15385) and flagellar assembly (*fliE*, *fliJ*, *fliP*, *fliA*, *fliK*) were upregulated, which may indicate that *Y. enterocolitica* modulates flagellar motility to respond to adverse environmental stress.

**Table 1:** Details of DEGs annotated by KEGG.

ko_Annotation	Genes Name	Function	Log2 FC
Oxidative phosphorylation	*nuoE*	NADH-quinone oxidoreductase subunit E	1.21
	*nuoF*	NADH-quinone oxidoreductase subunit F	1.49
	*nuoG*	NADH-quinone oxidoreductase subunit G	1.49
	*nuoH*	NADH-quinone oxidoreductase subunit H	1.76
	*nuoI*	NADH-quinone oxidoreductase subunit I	1.91
	*nuoJ*	NADH-quinone oxidoreductase subunit J	1.78
	*nuoK*	NADH-quinone oxidoreductase subunit K	1.44
	*nuoL*	NADH-quinone oxidoreductase subunit L	1.31
	*nuoM*	NADH-quinone oxidoreductase subunit M	1.04
	*nuoN*	NADH-quinone oxidoreductase subunit N	1.14
	*cyoE*	heme o synthase	1.26
	*cyoD*	cytochrome o ubiquinol oxidase subunit IV	1.1
	*cyoC*	cytochrome o ubiquinol oxidase subunit III	2.12
	*cyoB*	cytochrome o ubiquinol oxidase subunit I	1.23
	*cyoA*	cytochrome o ubiquinol oxidase subunit II	0.51
Fatty acid biosynthesis	*fabD*	malonyl-CoA acyl-carrier protein transacylase	−0.66
	*fabF*	3-oxyacyl-[acyl-carrier protein] synthetase II	−0.62
	*fabZ*	3-hydroxyacyl-[acyl-carrier-protein] dehydratase	−1
	*accC*	acetyl-CoA carboxylase biotin carboxylase subunit	0.96
ABC transporters	*metN*	D-methionine transport system ATP-binding protein	−1.2
	*cysP*	sulfate/thiosulfate transport system substrate-binding protein-sulfatel	−2.02
	*cysW*	sulfate/thiosulfate transport system permease protein	−2.58
	*yecC*	L-cystine ABC transporter ATP-binding protein *yecC*	−1.73
	RS14745	sugar ABC transporter substrate-binding protein	−3.18
	RS07200	amino acid ABC transporter permease	−2.84
	RS07205	amino acid ABC transporter permease	−2.04
	RS07210	ABC transporter substrate-binding protein	−2.19
	RS01410	sulfate ABC transporter substrate-binding protein	−2.02
	RS00015	methionine ABC transporter ATP-binding protein	−1.37
Cysteine and methionine metabolism	*cysK*	cysteine synthase A	−1.5
Cysteine and methionine metabolism	*cysM*	cysteine synthase *cysM*	−2.14
	*metK*	homoserine O-acetyltransferase	−1.98
	*mtnK*	S-methyl-5-thioribose kinase	−1.4
	RS12490	acireductone dioxygenase	−2.32
	RS12480	methylthioribulose 1-phosphate dehydratase	−1.89
Glutathione metabolism	*speF*	ornithine decarboxylase *speF*	−2.27
	*pepD*	beta-Ala-His dipeptidase	−1.05
Alanine, aspartate and glutamate metabolism	RS12470	amidohydrolase	−2.44
	*aspA*	aspartate ammonia-lyase	−1.25
	*carA*	glutamine-hydrolyzing carbamoyl-phosphate synthase small subunit	1.46
	*carB*	carbamoyl-phosphate synthase large subunit	1.02
Arginine biosynthesis	*argB*	acetylglutamate kinase	0.93
	*argC*	N-acetyl-gamma-glutamyl-phosphate reductase	0.93
	*argF*	ornithine carbamoyltransferase	1.17
Carbohydrate metabolism	*bcsA*	cellulose synthase (UDP-forming)	−0.66
	*glgC*	glucose-1-phosphate adenylyltransferase	−1.76
	*glgA*	starch synthase	−1.15
	*glgP*	glycogen phosphorylase	−0.66
	RS12080	sugar-binding transcriptional regulator	−1.28
Bacterial chemotaxis	RS19655	PAS domain-containing methyl-accepting chemotaxis protein	1.62
	RS15590	ABC transporter substrate-binding protein	1.52
	RS03695	methyl-accepting chemotaxis protein	0.77
	RS15385	chemotaxis protein	0.6
Quorum sensing	*lsrA*	autoinducer 2 ABC transporter ATP-binding protein *lsrA*	−1.88
	*lsrC*	AI-2 transport system permease protein	−1.37
	*lsrD*	autoinducer 2 ABC transporter permease *lsrD*	−0.87
	*lsrR*	transcriptional regulator *lsrR*	−0.8
	*yenR*	*luxR* family transcriptional regulator *yenR*	−0.34
	RS02520	ATP-binding cassette domain-containing protein	−1.29
	*oppA*	oligopeptide ABC transporter substrate-binding protein *oppA*	−0.67
	*rcsB*	transcriptional regulator *rcsB*	0.69
	*crp*	cAMP-activated global transcriptional regulator *CRP*	−0.36
	*flhC*	flagellar transcriptional regulator *flhC*	−0.96
	*flhD*	flagellar transcriptional regulator *flhD*	−0.41
Flagellar assembly	*fliE*	flagellar type III secretion system pore protein *fliP*	1.18
	*fliJ*	flagella biosynthesis chaperone *fliJ*	0.97
	*fliP*	flagellar type III secretion system pore protein *fliP*	0.77
	*flhA*	flagellar biosynthesis protein *flhA*	0.69
	*flgK*	flagellar hook-associated protein *flgK*	0.63

Although this study provides mechanistic insights into the inhibition of *Y. enterocolitica* biofilm formation by LA, several limitations remain. It remains to be determined whether the inhibitory effect of LA on *Y. enterocolitica* biofilm formation can be generalized to other strains. In addition, whether LA can exert antibacterial effects in the context of bacterial infection by modulating the host immune system remains to be determined. Therefore, in future work, we will use a broader range of clinical and environmental isolates to validate our findings and their general applicability, while establishing bacterial infection animal models to evaluate the anti-infective efficacy of LA and to systematically reveal the mechanisms by which it regulates host immune responses.

## 5. Conclusions

This study demonstrates that LA significantly inhibits biofilm formation by *Y. enterocolitica*. LA disrupts the structural and functional integrity of biofilms by compromising cell membrane integrity and reducing cellular adhesion. Additionally, LA can effectively inhibit mature biofilms and decrease the production of extracellular polysaccharides and proteins, which are crucial for biofilm stability. Observations with CLSM and FEG-SEM revealed that LA leads to a sparse biofilm structure and reduced bacterial aggregation. MTT assays further indicated that LA not only limits biofilm formation but also significantly inhibits the metabolic activity of bacteria within the biofilm. It also influences QS by regulating the levels of AHL signal molecules. At the molecular level, LA is involved in pathways related to energy metabolism, the QS system, bacterial chemotaxis, and flagellar assembly. By affecting flagellar motility and interfering with intercellular communication, it ultimately inhibits the formation of *Y. enterocolitica* biofilms. In summary, LA possesses antibiofilm properties, providing a theoretical basis for potential applications in microbial and biofilm control.

## Data Availability

The original contributions presented in this study are included in the article/Supplementary Material. Further inquiries can be directed to the corresponding authors.

## Supplementary Materials

The following supporting information can be downloaded at: https://www.mdpi.com/article/10.3390/microorganisms14030558/s1, Figure S1: The pH values of LA at different concentrations. Values are presented as the means of independent triplicate measurements (*n* = 3, representing biological replicates). Figure S2: Based on the CLSM images of *Y. enterocolitica* biofilms treated with LA, %PI-positive cells was quantitatively analyzed using ImageJ v.1.8.0. Values are presented as the means of independent triplicate measurements (*n* = 3, representing biological replicates). Figure S3: Relative viability of *Y. enterocolitica* after LA treatment. Values are presented as the means of independent triplicate measurements (*n* = 3, representing biological replicates). Figure S4: Effect of LA on the growth of *C. violaceum* CV026. Values are presented as the means of independent triplicate measurements (*n* = 3, representing biological replicates). Figure S5: RT-qPCR validation. Values are presented as the means of independent triplicate measurements (*n* = 3, representing biological replicates); Table S1: Primers used for RT-qPCR.
