# Supplementary material for: Mechanistic Insights into the Inhibition of Yersinia enterocolitica Biofilm Formation by Lipoic Acid
Source: Microorganisms. 2026 Feb 28;14(3):558. doi: 10.3390/microorganisms14030558 (PMC13029520; doi:10.3390/microorganisms14030558)
Supplement: Supplementary file 1 [file microorganisms-14-00558-s001.zip › microorganisms-4133096-supplementary.pdf]

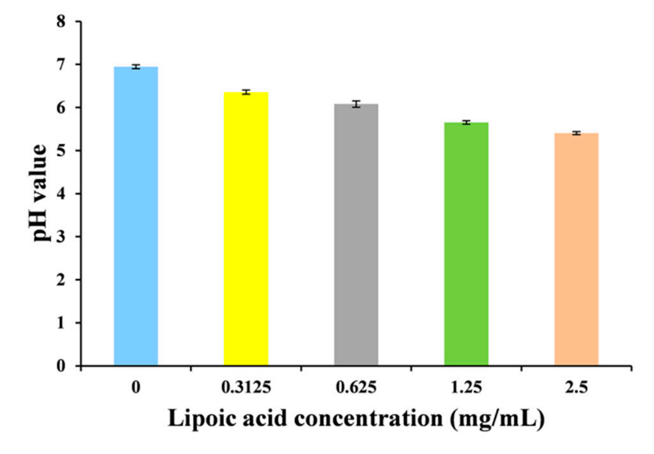

Figure S1. The pH values of LA at different concentrations. Values are presented as the means of independent triplicate measurements (n = 3, representing biological replicates).

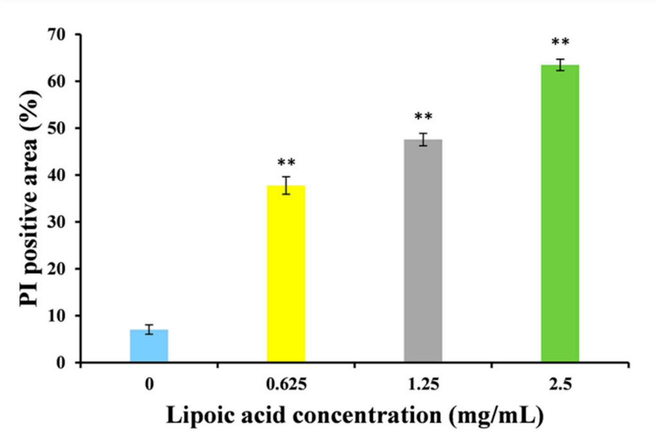

Figure S2. Based on the CLSM images of *Y. enterocolitica* biofilms treated with LA, %PI-positive cells was quantitatively analyzed using ImageJ. Values are presented as the means of independent triplicate measurements (n = 3, representing biological replicates). **p* < 0.05, ***p* < 0.01.

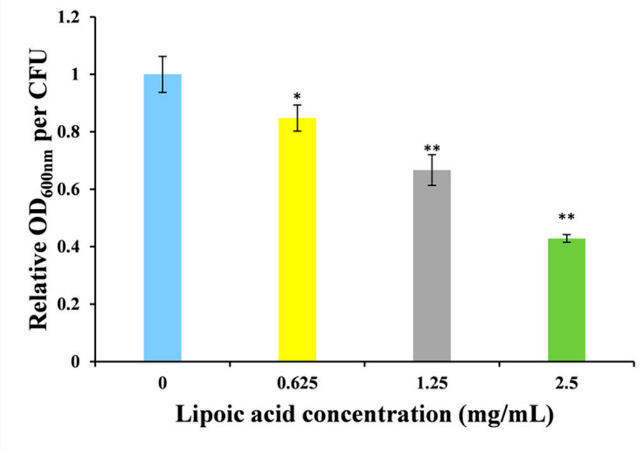

Figure S3. Relative viability of *Y. enterocolitica* after LA treatment. Values are presented as the means of independent triplicate measurements (n = 3, representing biological replicates). *$p < 0.05$, **$p < 0.01$.

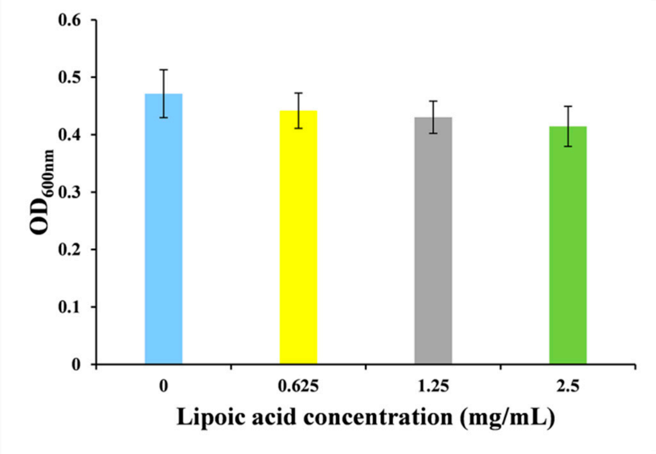

Figure S4. Effect of LA on the growth of *C. violaceum* CV026. Values are presented as the means of independent triplicate measurements (n = 3, representing biological replicates). *$p < 0.05$, **$p < 0.01$.

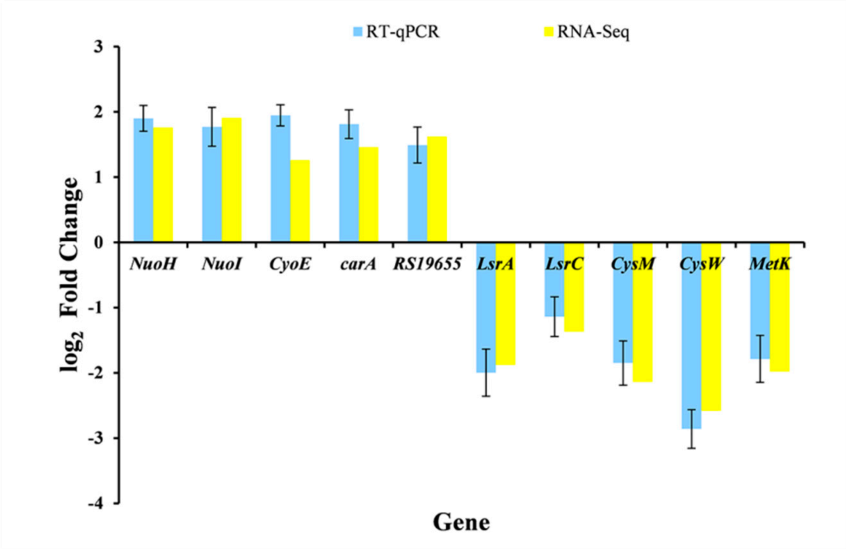

Figure S5. RT-qPCR validation. Values are presented as the means of independent triplicate measurements (n = 3, representing biological replicates).

Table S1. Primers used for RT-qPCR.

Gene	Primer	Sequence (5'-3')
NuoH	*NuoH*-F	TTTACCTCGCTGCTGCTCTC
	NuoH-R	TATTACTCGACCAACCGGCG
NuoI	*NuoI*-F	CTTCATGCCTTCCACAAGCG
	NuoI-R	CACACAGGTTACACGCAACG
CyoE	*CyoE*-F	ACCTTAAGTGGCTATGCGGG
	CyoE-R	TACGCTGTCATTGGTCGCTT
carA	*carA*-F	CTGCAAGGCAGTTGGACTCT
	carA-R	CGAGCATGCGCAGGATATTG
RS19655	*RS19655*-F	CAGCTCCGTTTATTGCTCGC
	RS19655-R	GCCCACCAGGGTAATCAGAG
LsrA	*LsrA*-F	GTGAAATTCTTGGCCTGGCG
	LsrA-R	GGTGATGTCGCACTCTTCCA
LsrC	*LsrC*-F	TGGTGATGCTAACCCGCAAT
	LsrC-R	GCCAACCAATAGCGCAAACA
CysM	*CysM*-F	CCGAGGGCAGTAGTATTCCG
	CysM-R	CTGTTCGGCTGAAATCTGCG
CysW	*CysW*-F	CCGGTGATGATGAGTCAGGG
	CysW-R	CACCACGCCATACAGCAATG
MetK	*MetK*-F	CGAGTTGCCTGCGAGACTTA
	MetK-R	GATTTCGCGCACAGTACGAC
16s rRNA	*16s rRNA*-F	CTAAGCGGGTGTTTCAGGCA
	16s rRNA-R	ATCGGTAATCCTGGCGGCAA
